# Integrated analysis strategy of genome-wide functional gene mining reveals *DKK2* gene underlying meat quality in Shaziling synthesized pigs

**DOI:** 10.1186/s12864-023-09925-x

**Published:** 2024-01-04

**Authors:** Shuaihan He, Yubei Wang, Yabiao Luo, Mingming Xue, Maisheng Wu, Hong Tan, Yinglin Peng, Kejun Wang, Meiying Fang

**Affiliations:** 1 State Key Laboratory of Animal Biotech Breeding, College of Animal Science and Technology, China Agricultural University, Beijing, 100193 China; 2 Sanya Institute of China Agricultural University, Sanya, 572025 China; 3 Xiangtan Bureau of Animal Husbandry and Veterinary Medicine and Aquatic Product, Xiangtan, 411102 China; 4 Hunan Institute of Animal & Veterinary Science, Changsha, 410131 China; 5 College of Animal Science and Technology, Henan Agricultural University, Zhengzhou, 450002 China

**Keywords:** Shaziling pigs, Whole genome sequencing, Selective sweep, IBD analysis, *DKK2*, Meat quality

## Abstract

**Background:**

Shaziling pig is a well-known indigenous breed in China who has superior meat quality traits. However, the genetic mechanism and genomic evidence underlying meat quality characteristics of Shaziling pigs are still unclear. To explore and investigate the germplasm characteristics of Shaziling pigs, we totally analyzed 67 individual’s whole genome sequencing data for the first time (20 Shaziling pigs [S], 20 Dabasha pigs [DBS], 11 Yorkshire pigs [Y], 10 Berkshire pigs [BKX], 5 Basha pigs [BS] and 1 Warthog).

**Results:**

A total of 2,538,577 SNPs with high quality were detected and 9 candidate genes which was specifically selected in S and shared in S to DBS were precisely mined and screened using an integrated analysis strategy of identity-by-descent (IBD) and selective sweep. Of them, dickkopf WNT signaling pathway inhibitor 2 (*DKK2)*, the antagonist of Wnt signaling pathway, was the most promising candidate gene which was not only identified an association of palmitic acid and palmitoleic acid quantitative trait locus in PigQTLdb, but also specifically selected in S compared to other 48 Chinese local pigs of 12 populations and 39 foreign pigs of 4 populations. Subsequently, a mutation at 12,726-bp of *DKK2* intron 1 (g.114874954 A > C) was identified associated with intramuscular fat content using method of PCR–RFLP in 21 different pig populations. We observed *DKK2* specifically expressed in adipose tissues. Overexpression of *DKK2* decreased the content of triglyceride, fatty acid synthase and expression of relevant genes of adipogenic and Wnt signaling pathway, while interference of *DKK2* got contrary effect during adipogenesis differentiation of porcine preadipocytes and 3T3-L1 cells.

**Conclusions:**

Our findings provide an analysis strategy for mining functional genes of important economic traits and provide fundamental data and molecular evidence for improving pig meat quality traits and molecular breeding.

**Supplementary Information:**

The online version contains supplementary material available at 10.1186/s12864-023-09925-x.

## Background

Pork is the most widely consumed meat worldwide due to its delicacy especially in China. Meat quality is the main attribute that affects consumers’ preference for meat consumption [1]. The major meat quality traits are represented by the pH value, drip loss, meat color, tenderness, and intramuscular fat (IMF) content. A relative higher IMF content could lead to better meat flavor because lipids, the important flavor precursors, produce volatile compounds via lipid oxidation [2]. The IMF is primarily composed of triglycerides and phospholipids, both of which produce fatty acids via hydrolysis [3]. Fatty acids are closely related to meat quality such as edibility, juiciness, tenderness, and flavor [4, 5].

Compared to western commercial pig breeds, Chinese indigenous pig breeds normally have a slower growth rate and lower lean meat content, but they are superior in meat quality, marbling grade and IMF content, which arouses researcher’s interest. Of them, Shaziling pig (S), one of the excellent indigenous pig breeds in Hunan province, has superior meat quality characteristics compared to most other pigs [6, 7]. It was also reported that predominant fatty acids identified in the longissimus thoracis muscle of Shaziling pigs were C16:0, C16:1, C18:0, C18:1n9c, and C18:2n6c, which might be promising indicators for better meat quality [8]. However, it remains unclear how the excellent meat quality traits of Shaziling pigs develop and there is only few research on Shaziling pigs to provide genome evidence and mine functional genes relevant to meat quality traits for further breeding improvement.

The development of high-throughput whole genome sequencing (WGS) technology and bio-information technology expanded our ability to study the genetics of farm animals. Livestock genomes can be analyzed by several statistical measures and approaches to reveal specific regions under selection, giving an in-depth understanding of how genomic variation has been shaped and advantageous characteristics have evolved through strong artificial and natural selection [9–11]. Relevant study of WGS has become quite common in swine genomic researches and plenty of them can provide insights into the biological mechanisms that lead to morphological differentiation, specialized production performances and, in some cases, disease resistance and resilience [12–16], which has great economic value and significance in livestock and poultry breeding improvement.

Selection will usually tend to increase identity-by-descent (IBD) among individuals in a population [17] and therefore several authors have developed methods that are able to infer IBD tracts shared between pairs of individuals from outbred populations without any pedigree information, using dense genotyped data such as SNP chip data [18–20]. The original purpose of these methods was to identify regions of the genome-harboring disease loci and then these methods was used to study the genetic history of the populations [21]. IBD analysis also can provide a new approach for detecting very recent and strong selection in the genome, which is not only selection acting on a new allele, but also selection acting on standing variation [17]. Majority of swine IBD research focused on precise detection and refined localization of quantitative trait loci (QTL) such as growth, fatness, meat quality or disease [22–25]. It was reported that IBD analysis was used to estimate the actual pedigree relationships of pigs and control inbreeding [26–29]. Previous IBD study of European and Asian pig genomes also revealed fine-scale haplotype structure of different populations and reconstructed demographic histories [30].

Here, we conducted a comprehensive whole-genome analysis of 67 individuals from the Shaziling line pigs and identified candidate genes associated with meat quality traits using an integrated analysis approach combining selective sweep and IBD analysis. We further explored and elucidated the biological functions of the candidate gene *DKK2*. These findings offer novel insights into the identification of functional genes relevant to economically significant traits and facilitate molecular breeding efforts for improving the Shaziling pig breed.

## Materials and methods

### DNA sample collection and resequencing

We totally resequenced 55 samples which were all from livestock breeding station of Xiantan city (Hunan province, China), including 20 Shaziling pigs (S), 10 Berkshire pigs (BKX), 20 Dabasha pigs (DBS) and 5 Basha pigs (BS) in this study. We used TIANamp Genomic DNA Kit (DP304, TIANGEN) to extract genomic DNA from pig ear tissue. Quality, integrity, and concentration of extracted DNA were detected by 1.5% agarose gel electrophoresis and Thermo Scientific Nanodrop 2000 microvolume spectrophotometer. All qualified genomic DNA was resequenced using Illumina HiseqTM 2500 sequencing system. The Illumina DNA libraries (paired-end, 2*150 bp) were constructed for 55 pig samples using at least 1 μg genomic DNA with standard protocols and 2732.22 Gb raw data were generated in 10 × or 30 × sequencing depth. Further, we downloaded 11 Yorkshire pigs and 1 Warthog whole genome data from European Nucleotide Archive (ENA) database and totally we got 517.23 Gb raw data.

### Read alignment and variant calling

In order to facilitate better reads mapping, quality control was carried out by FastQC (version: 0.12.1) and MultiQC (version: 1.9) [31], and then all raw reads were filtered and trimmed using Trimmomatic (version: 0.39) [32] if any of the following criteria were met:

1. Reads with adapter sequence and poly-N.
2. Reads whose low-quality base ratio (base quality less than or equal to 5) is more than 50%.
3. reads whose unknown base (“N” base) ratio is more than 10%.

After quality control, filtered paired-end reads were aligned individually to the Swine reference genome Sus scrofa 11.1 using Burrows-Wheeler Aligner [33] (BWA version: 0.7.17-r1198) with following default parameters. Subsequently, SAMtools [34, 35] (version: 0.1.19-44428cd) was used to convert the file format from *.sam to *.bam and filter the unmapped and nonunique reads. The procedure of “Mark Duplicates” and “SortSam” was then performed and the aligned files (*.bam) of 67 pigs were used for SNP detection using GATK (Version: 4.011) [35] and filtered with following standards [36]:

1. variant confidence/ quality by depth (QD) > 2;
2. Phred-scaled p-value using Fisher’s Exact Test to detect strand bias (FS) < 60;
3. the Root Mean Square of the mapping quality of the reads across all samples (MQ) > 40;
4. Z-score from the Wilcoxon rank sum test of Alt vs. Ref read MQs (MQRankSum) > -12.5;
5. Z-score from the Wilcoxon rank sum test of Alt vs. Ref read position bias (ReadPosRankSum) > -8;
6. vcftools –max-missing 0.8.

The variants after filtering were processed for gene-based or region-based annotations using SnpEff (version: 4.3t) [37]. Corresponding annotation file were downloaded from Ensembl to assist SNPs annotation. According to the region and function, SNPs were classified into nine common SNP categories.

### Population structure analysis

As for construction of phylogenic tree, we filtered all genotyped variants for 67 pigs and converted filtered variants files (*.vcf) into PLINK format files (*.ped and *.map) using PLINK (version: 1.90b4.1) [38]. Second, the IBS distance matrix between individuals was calculated by PLINK using the resulting 1,173,001 SNPs. Finally, based on the distance matrix, phylogenic tree was constructed by TreeBeST [39] in the method of neighbor-joining (NJ), bootstrap was calculated totally 100 times. We performed the PCA with PLINK format files (*.ped) which we got from previous step using PLINK.

The construction of population genetic structure used ADMIXTURE (version 1.30) [40], which estimated the specific admixture proportions among 5 populations using all high-density SNPs data. Eleven scenarios (ranging from K = 2 to K = 12) were selected for genetic clustering with cross-validation (CV) error procedure. Then the results were plotted using R.

Levels of linkage disequilibrium (LD) for pig populations were assessed by genotype correlation coefficient (r^2^) between any two loci (within and between different chromosomes) using PopLDdecay (version: 3.42) [41]. LD decays were plotted using an in-house self-script and R packages.

### Selective sweep, identity by descent and functional enrichment candidate genes

In order to detect the regions with significant selective signatures of S, we calculated the Fst and θπ ratios to measure the population selective differentiation (S vs Y, S vs B) using in-house self-script and VCFtools (version: 0.1.17) [42] with a 200-kb sliding-window with 100-kb increments. We regarded the top 5% values for both Fst and log_2_θπ as the strong selected region, and the “intersect” subcommand of BEDTools (version: 2.30.0) were used to get the overlapping regions of Fst and θπ results. Intersections were annotated using SnpEff.

Based on the high-quality SNPs of each population, identity by descent was analyzed using BEAGLE (version: 4.1) [43] with the default parameters except parameter “ibd = true” changed to get the IBD interval of pairwise comparison between populations. We selected the top 50% of LOD score as the representative IBD interval of each population. Then, based on the pedigree genetic relationship, the IBD interval of the progeny population transmitted from its parents were obtained using the “intersect” subcommand of BEDTools.

We took the intersection results of selective sweep and IBD analysis as candidate regions. The biological function of genes within candidate regions was annotated by analyzing Gene Ontology and KEGG using DAVID. Benjamini–Hochberg false discovery rate (FDR) was used for correcting the P values. Candidate genes were also searched in the Animal QTLdb (https://www.animalgenome.org/cgi-bin/QTLdb/SS/index) to learn about its function. Furthermore, the most promising candidate *DKK2* gene was detected in 87 individuals of 16 populations using the method of selective sweep mentioned above with the same parameters to identify the gene diversity of *DKK2* in different populations of foreign and Chinese local pigs.

### Genotypic and phenotypic analysis

The research animal in this part consists of 352 pigs from 21 different populations. Of them, 299 pigs’ (from 15 different populations) ear samples were collected in 75% alcohol and stored at -20℃ from corresponding conservation farm across the China and then used for DNA extraction. *DKK2* was genotyped by PCR–RFLP using the reaction condition of the 5-min pre-denaturation at 95℃, 30-s denaturation at 95℃, 30-s annealing at 59℃, 35-s extension at 72℃ and 10-min final extension at 72℃. The primers of PCR–RFLP saw in additional file 1: Table S1. The results of PCR amplification were digested by restriction endonuclease BspHI (NEW ENGLAND BioLabs) with the reaction condition of 60-min incubation at 37℃ and 20-min inactivation at 65℃ and finally visualized on 1.5% agarose gels. All 352 samples of 21 pig populations’ intramuscular fat content data were collected from *Chinese Livestock and Poultry Genetic Resources: Pig Records.* For pigs at the end of the fattening period, fresh samples of the longest muscle on the back of the pig at the 2–3 ribs from the end were collected after slaughter. The intramuscular fat was determined using the chemical analysis method (Soxhlet extraction method). The association studies between genotype and phenotype were performed with the regression analysis of GenABEL package in R.

### Quantitative real-time PCR analysis

The total RNA was isolated from pig adipose tissues or adipocytes using TRIzol® reagent (Invitrogen) and subjected to reverse transcription using the FastKing RT Kit (KR118, TIANGEN) according to the manufacturer’s instructions. Quantitative real-time PCR (qPCR) was measured with the talent qPCR (FP209, TIANGEN) using the Bio-Rad CFX96 Real-Time PCR Detection System. The amplification cycles were carried out at 95°C for 10 s, 60°C for 20 s, 72°C for 20 s. After 39 cycles, the CT value was quantified. The total amount of mRNA was normalized to endogenous β-actin mRNA. And the comparative threshold cycle (2^−△△ct^) method was used to calculate the relative expression. The primer sequences for quantitative PCR (Additional file 1: Table S1) were designed using Primer Premier 5.0 software (Premier Biosoft International, Palo Alto, CA) and then synthesized by Sangon Biotech (Shanghai, China). *DKK2* tissues expression profile was measured by semi-qPCR with 28 amplification cycles.

### Plasmid constructs and cell transfection

*DKK2* CDS (XM_003129269) was amplified by PCR and subcloned into pCDNA 3.1 + vector using SE seamless cloning and assembly kit (ZC232, Zomanbio) according to the manufacturer’s instructions. siRNA sequences and shRNA vector (Additional file 1: Table S2) were synthesized by GenePharma (Jiangsu, China) and we identified their interfere effect by means of RT-qPCR after 48 h transfection to choose the optimum shRNA (Additional file 2: Fig. S1A) and siRNA (Additional file 2: Fig. S1B). Before transfection, porcine preadipocytes and 3T3-L1 cells were seeded into 12-well plates at 5.0 × 10^5^ cells/well. After overnight attachment (about 90% confluence), transfections were performed using lipofectamine 3000 (Invitrogen) according to the manufacturer’s instructions.

### Cell culture and differentiation

Immortalized porcine preadipocytes were got from our previous research [44], while 3T3-L1 cells was purchased from American Type Culture Collection (ATCC). The porcine preadipocytes were maintained in Dulbecco’s modified Eagle’s medium/nutrient mixture F-12 (DMEM/F12, Gibco) with 100U/ml penicillin (Gibco), 100U/ml streptomycin (Gibco) and 10% fetal bovine serum (Gibco) at 37℃ in a humidified atmosphere of 95% air and 5% CO_2_. To convert porcine preadipocytes to adipocytes, the cells inoculated in 12-well plates and maintained in DMEM/F12 complete medium until they reached 100% confluence. Then porcine preadipocytes were incubated in DMEM/F12 complete medium supplemented with 0.25 mM 3-isobutyl-1-methylxanthine (IBMX, Sigma), 100 nM rosiglitazone (Sigma), 1μM dexamethasone (Sigma), and 5μg/ml insulin (Sigma) for 48 h adipocyte differentiation. After that, adipocyte differentiation medium was replaced by DMEM/F12 complete medium containing 10 mg/ml insulin. The cells were harvested every 2 days after transfection for further research (porcine preadipocytes lasted to 12th day after differentiation while 3T3-L1 lasted to 8th day). As for 3T3-L1 cells culture and differentiation, the process and method are basically same except for three differences: (1) using DMEM rather than DMEM/F12; (2) changing into adipocyte differentiation medium on 2nd days after 100% confluence; (3) Not putting rosiglitazone into differentiation medium.

### Oil-red O staining and triglyceride content detection

The differentiated porcine preadipocytes and 3T3-L1 cells were stained with oil-red O as Zhong previously described [45]. The cells were washed twice with phosphate-buffered saline (PBS, Gibco) and fixed by 4% formaldehyde at room temperature for 30 min. Next, the formaldehyde was discarded, and the cells were washed twice again with PBS. Subsequently, fresh oil-red O solution (Sigma) was added and incubated at room temperature for 30 min. After that, the stained cells were microscopically examined. To quantity the amount of lipid contents, cells were incubated with 100% isopropanol for 5 min and the solvent was measured for the absorbance at 510 nm using BioTek Synergy HT Plate Reader.

### Fatty acid synthetase enzyme-linked immunosorbent assay

Before detecting the FAS components in cells, cell suspension was diluted with PBS to the cell concentration reaching about 1 million/ml. Cell internal components were released by repeatedly freezing at liquid nitrogen and thawing at 37°C (at least 3 times) and subsequently were collected through 3,000 rpm centrifuging for 10 min. FAS ELISAs about porcine preadipocytes and 3T3-L1 cells were performed using FAS ELISA Kit (Mlbio) according to the manufacturer’s instructions and then measured at 450 nm using a spectrophotometer. Standards curve of optical density versus FAS concentration was produced by calibration standards. The concentration of FAS in the samples was then determined by comparing the O.D. of the samples to the standard curve.

### Statistical analysis

All data presented were obtained from at least three independent experiments and are expressed as the mean ± SEM. Differences between groups were analyzed by using Student’s t-test associated with Excel software. Statistical significance was set to ^∗^*P* < 0.05, ^∗∗^*P* < 0.01, and ^∗∗∗^*P* < 0.001.

## Results

### Overview of sequencing and detected variants

**Fig. 1:**
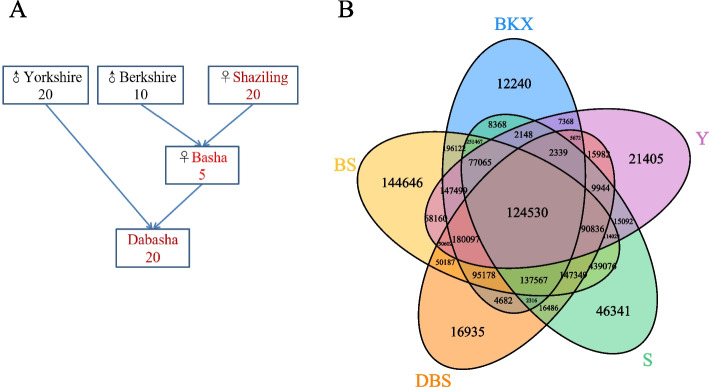
The pedigree of Dabasha crossbred line and SNPs of each population.** A** The pedigree of Dabasha crossbred line and the number of analyzed pigs. **B** Venn diagram of exclusive and common SNPs of each population

We performed genome resequencing on 55 domestic pigs, representing 4 different breeds of the Dabasha crossbred line (Fig. 1A), at sequencing depths of 10 × and 30x (Additional file 1: Table S3). A total of 2715.11Gb of high-quality paired-end sequence data were obtained, with an average mapping rate of 98.40% to the latest reference genome. These datasets were combined with the published whole-genome sequencing data of 11 Yorkshire pigs and 1 Warthog, resulting in the download of 468.83Gb of high-quality sequencing data from the database, with an average depth of 13.84x (Additional file 1: Table S4).

A total of 2,538,577 high-quality single-nucleotide polymorphisms (SNPs) were detected using GATK in the entire population, including the Warthog (Additional file 1: Table S5). Among these SNPs, 861 were newly identified (approximately 0.03392%), potentially indicating their presence at lower frequencies or their specificity to the Shaziling population, which may explain why they were not previously detected. Further annotation of these variants revealed that the SNPs were most prevalent in intergenic regions (approximately 52.996%), followed by intronic, UTR, upstream and downstream, exonic, and splicing site regions, in line with previous research findings [12]. In exonic regions, we identified 16,767 potential functional genetic variations, including 10,587 synonymous SNPs, 6,119 nonsynonymous SNPs, 44 stop gain SNPs, and 17 stop loss SNPs. These potential functional SNPs represent valuable genetic resources for further exploration of the genetic structure and selective signatures in the population. Additionally, we obtained the number and Venn diagram of SNPs shared by each population (Fig. 1B). We identified 124,530 common SNPs and a total of 241,567 unique SNPs for each population (Additional file 1: Table S6). These SNPs can provide valuable insights for further research on selective sweeps and identity-by-descent (IBD) analysis to explore germplasm characteristics and similarities.

### Genetic diversity and population structure

**Fig. 2:**
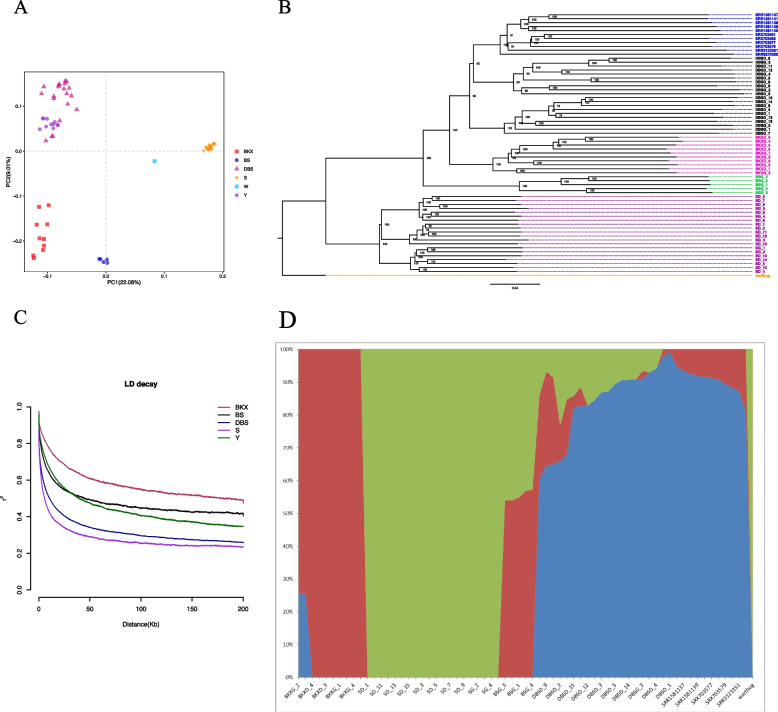
Population genetic analysis of Dabasha crossbred line. **A** Principal component analysis of all sample individuals. The fraction of the variance explained is 22.08% and 9.01% for PC1 and PC2, respectively. **B** Neighbor-Joining phylogenetic tree of all sample individuals. 0.04 in the bottom of the tree represented same branch length’s nucleotide mutation was 4% (Each of 100nts had 4 mutations on average) and the number at nodes indicated mostly 100 bootstrap supports. **C** Linkage disequilibrium decay of five populations measured by r^2^. **D** When K = 3, population structure of all individuals. Red represents the genetic background from B, while green represents S and Blue represents Y

To clearly demonstrate the population structure of pigs in our study, we utilized high-density SNP data to construct a phylogenetic tree and perform principal component analysis (PCA). The PCA analysis of 67 pigs resulted in distinct genetic clusters that aligned with their main characteristics (Fig. 2A). Notably, on the PC1 dimension, the PCA analysis revealed a clear separation between western commercial pigs (Y and BKX) and the China indigenous pig (S), reflecting their differences in artificial selection and geographic origin. DBS and Y exhibited proximity in the two-dimensional space, indicating a closer genetic relationship between DBS and Y. These findings were further supported by the phylogenetic tree constructed using the neighbor-joining method based on the same set of SNPs (Fig. 2B). During the analysis of linkage disequilibrium (LD), we observed that the LD decay rate was the fastest in the China indigenous pig (S) and the slowest in the BKX (Fig. 2C). This suggests that S may possess a greater abundance of genetic diversity, potentially due to experiencing lower selection pressure.

To analyze the population structure, we applied the genetic co-ancestry theory [46] and constructed a structure plot with K values ranging from 2 to 12 (Additional file 2: Fig. S2). Based on cross-validation results (Additional file 1: Table S7), the K value of 3 (Fig. 2D) had the lowest cross-validation error (0.33874) and demonstrated the most reliable admixture result. Three distinct clusters were clearly observed, indicating the genetic influence of the parent breeds in the DBS three-way crossbred line.

### Integrated Co-analysis of genome-wide selective sweep and identity by descent

**Fig. 3:**
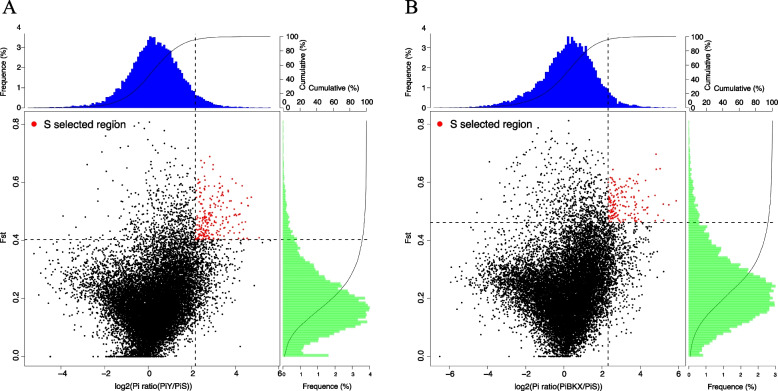
Genome-wide regions with selection sweep signals.** A** S selected region compared to Y. The red spots represent intersection of top 5% Fst and log_2_ (θπ ratios) and we calculated the frequency of selected region in different Fst and log_2_ (θπ ratios) value. **B** S selected region compared to BKX

The results of the genetic diversity analysis revealed significant population genetic differences in the S pig compared to others. To further investigate the genomic evidence contributing to the unique features of S, we conducted a comparison of the genomic selective signatures between S and other pig populations, particularly Y (Fig. 3A) and BKX (Fig. 3B) and identified the intersection of the two results. We calculated the genetic differentiation (Fst) and nucleotide diversity difference (θπ) for pairwise comparisons among populations using a sliding window approach of 200-kb along the genome. Genes under selection were identified based on the intersection of the top 5% Fst values and the log2 (θπ ratios). After intersecting the selected regions of S compared to Y and S compared to BKX, a total of 111 candidate genes unique to S were identified (Additional file 1: Table S8). Enrichment analysis revealed that most of these genes were associated with retinol metabolism, tyrosine metabolism, fatty acid degradation, and glycolysis/gluconeogenesis pathways. Additionally, the molecular functions of these genes were found to be primarily related to oxidative metabolism and molecular binding (Additional file 1: Table S9 and 10).

To investigate the transmission model of genes involved in S characteristic traits from parental to filial generations, we utilized pedigree information and identified shared identity-by-descent (IBD) blocks from S to BS to DBS (S-BS-DBS) using Beagle software and SNP data. We considered the top 50% of LOD scores as representative IBD intervals for each population. This analysis resulted in a total of 278 IBD blocks (spanning 19.8M in length) and 229 genes shared between S and DBS (Additional file 1: Table S11).

We observed that some pathways overlapped with those identified in the selective sweep analysis, such as fatty acid degradation (Additional file 1: Table S12 and 13). Therefore, we performed an intersection analysis between the IBD and selective sweep results. As a result, we identified 9 candidate genes: *LOC102165777*, *LOC106504669, EXOC1*, *LOC100620408*, *CEP135*, *LOC102158358*, *LOC110262110*, *LOC110262177*, and *DKK2.* Of particular interest, *DKK2* emerged as the most promising gene, as we observed its association with the content of palmitic acid and palmitoleic acid in the pig QTL database. Based on this finding, we focused our attention on the *DKK2* and investigated its selection signal in 87 individuals from 16 populations, including 39 foreign pigs from 4 populations and 48 Chinese local pigs from 12 populations (Additional file 1: Table S4 and 14). Comparing *DKK2* in other Chinese local pigs, we found that the θπ ratio (S/other Chinese local pigs) was 0.5472, while the θπ ratio (Foreign/Chinese local pigs) was 0.5013 (Additional file 1: Table S15 and 16). This indicates that the *DKK2* was highly selected in both S and foreign pigs compared to other Chinese local pigs. Additionally, we discovered significant haplotype differences between S and foreign pigs (Additional file 2: Fig. S3). The Fst value between foreign and Chinese local pigs was 0.485, suggesting that the *DKK2* was subjected to different selective pressures and served different purposes in Chinese and foreign pigs.

### Association of genomic variant at *DKK2* gene with intramuscular fat content

**Fig. 4:**
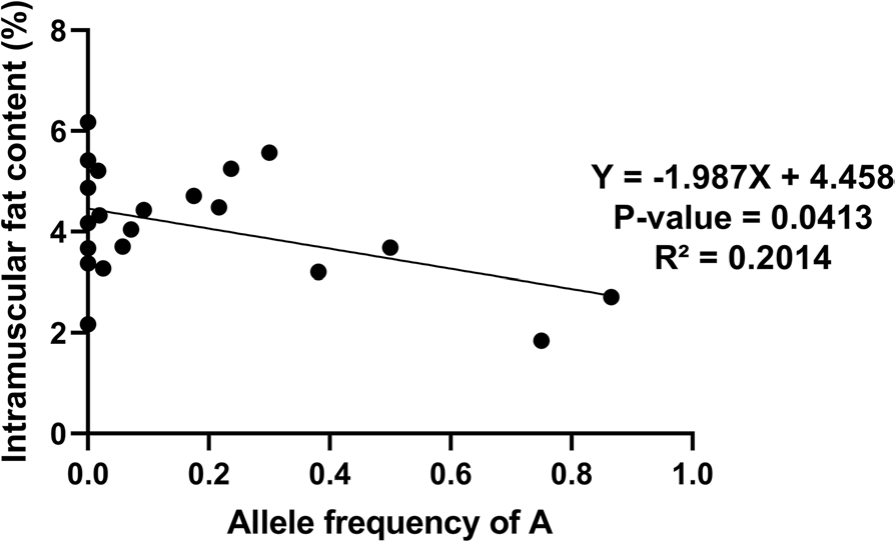
The linear regression of allele frequency as X and intramuscular fat content (%) as Y

The porcine *DKK2* is situated on chromosome 8 and comprises of four exons and three introns. To identify the potential single nucleotide polymorphism (SNP) site associated with S characteristic traits, we thoroughly examined all variants within the *DKK2* using our sequencing data (Additional file 1: Table S17). Notably, our sequence analysis of *DKK2* revealed a mutation: A > C at the 12,726-bp position of intron 1 (g.114874954 A > C). While the reference genome displays an A, the S population predominantly exhibits a C (Additional file 1: Table S18). We conducted PCR–RFLP genotyping on 352 individuals from 21 representative pig populations (Table 1). In all Chinese indigenous pigs, we observed that the C allele was dominant, whereas the A allele frequency was higher only in the Duroc population. Furthermore, the results of the Chi-square (χ^2^) test demonstrated that the SNP genotype was consistent with the Hardy–Weinberg equilibrium. Association analysis was performed between the population SNP genotyping results and average intramuscular fat content of 21 pig populations. The results revealed that there was a trend that average intramuscular fat content of pig population and its C allele frequency increased together. Regression analysis with the frequency of A allele as X and the intramuscular fat content as Y was then performed (Fig. 4). The linear regression model was successfully constructed: Y = -1.987X + 4.458 (*P* = 0.0413, R^2^ = 0.2014), A allele frequency of this SNP site is significantly negatively correlated with intramuscular fat content.

**Table 1:** Genotyping results and allelic frequencies of *DKK2* gene in 21 different populations

Breed	Number	Average Intramuscular Fat Content (%)	Genotype			Allele Frequencies		χ^2^(P)
			CC	AC	AA	C	A	
Min	19	5.25 ± 0.12	11	7	1	0.763	0.237	0.007 (0.997)
Zaozhuang heigai	30	4.48	18	11	1	0.783	0.217	0.193 (0.908)
Shaziling	20	3.27 ± 1.07	19	1	0	0.975	0.025	0.013 (0.993)
Daweizi	5	5.57 ± 0.15	2	3	0	0.700	0.300	0.918 (0.632)
Ningxiang	7	4.04 ± 0.42	6	1	0	0.929	0.071	0.041 (0.979)
Jinhua	26	3.7 ± 0.43	23	3	0	0.942	0.058	0.097 (0.952)
Xiaomeishang	21	4.87	21	0	0	1.000	0	-
Lantang	30	5.21 ± 0.33	29	1	0	0.983	0.017	0.009 (0.996)
Putian	27	4.43 ± 0.09	22	5	0	0.907	0.093	0.281 (0.869)
Enshi	26	4.32 ± 0.68	25	1	0	0.981	0.019	0.010 (0.995)
Neijiang	7	5.42	7	0	0	1.000	0	-
Tibet	20	4.71 ± 0.59	13	7	0	0.825	0.175	0.900 (0.638)
Yorkshire	38	3.20 ± 0.13	15	17	6	0.618	0.382	0.103 (0.950)
Berkshire	10	3.68 ± 0.99	3	4	3	0.500	0.500	0.400 (0.819)
Landrace	10	1.84 ± 0.09	1	3	6	0.25	0.75	0.400 (0.819)
Jiangquhai	4	6.18 ± 1.40	4	0	0	1	0	-
Leping	2	2.17 ± 0.46	2	0	0	1	0	-
Chuanxiang	2	4.17 ± 0.45	2	0	0	1	0	-
Rongchang	5	3.67 ± 2.02	5	0	0	1	0	-
Wannan	2	3.37 ± 0.13	2	0	0	1	0	-
Duroc	41	2.71 ± 0.08	0	11	30	0.134	0.867	0.984(0.611)
Total	352		230	75	47

### Molecular and cellular identification of *DKK2* gene function

Considering that *DKK2* functions as a negative regulator of the Wnt/beta-catenin signaling pathway and has been associated with fatty acid QTLs, we aimed to investigate its potential role in adipogenesis, particularly in fatty acid synthesis. Initially, we assessed the mRNA expression level of *DKK2* in various tissues. Semi-quantitative analysis of tissue expression profiles in Duroc pigs and S revealed that *DKK2* exhibited specific expression in the muscle, abdominal fat, and backfat (Additional file 2: Fig. S4A), indicating its potential significance in adipose tissues. Furthermore, when compared to Duroc pigs, we observed an up-regulation of *DKK2* expression in both types of adipose tissues in S, as confirmed by RT-qPCR analysis (Additional file 2: Fig. S4B).

**Fig. 5:**
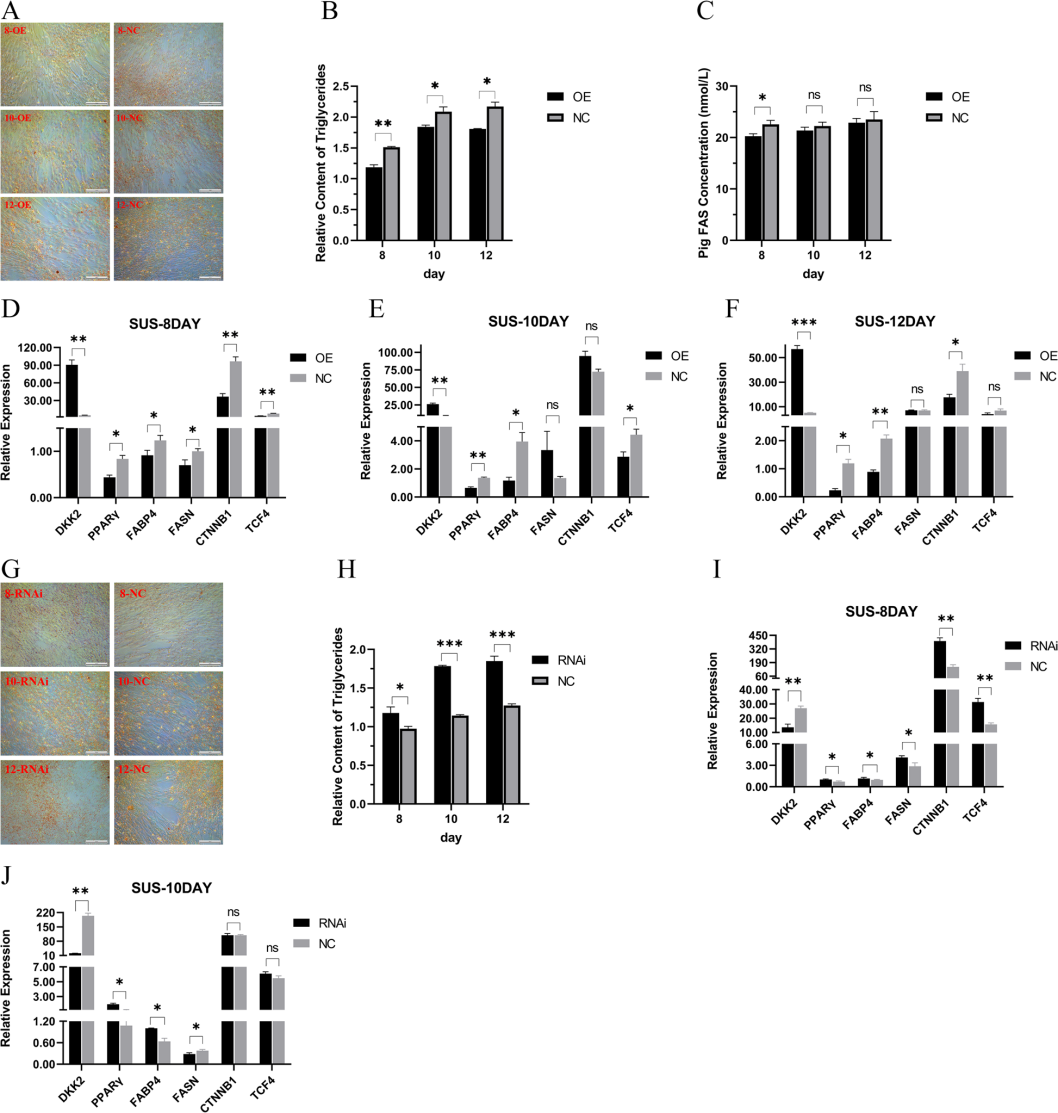
The overexpression and RNA interfere of *DKK2* gene on immortalized porcine preadipocytes. **A** Lipid droplet accumulation of overexpression and negative control group on 8th, 10th and 12th day after differentiation. **B** Relative content of triglycerides of overexpression and negative control group on 8th, 10th and 12th day after differentiation. **C** Overexpression and negative control group’s concentration of fatty acid synthase on 8th, 10th and 12th day after differentiation. The mRNA levels of overexpression group and negative control group on 8th (**D**), 10th (**E**), 12th (**F**) day after differentiation. **G** Lipid droplet accumulation of RNAi and negative control group on 8th, 10th and 12th day after differentiation. **H** Relative content of triglycerides of RNAi and negative control group on 8th, 10th and 12th day after differentiation. The mRNA levels of RNAi group and negative control group on 8th (**I**), 10th (**J**) day after differentiation

Based on these findings, we proceeded to validate our hypothesis using immortalized porcine preadipocytes and 3T3-L1 cells through overexpression and silencing of the *DKK2*. Initially, we examined the expression of *DKK2* mRNA during induced differentiation. The highest expression levels of *DKK2* in both porcine preadipocytes and 3T3-L1 cells were observed on the 8th day, indicating the involvement of *DKK2* in the middle or late stages of adipocyte differentiation (Additional file 2: Fig. S5). As the ability to accumulate lipid droplets is a crucial characteristic of mature adipocytes, we investigated the impact of *DKK2* overexpression on lipid droplet aggregation using oil red O staining and triglyceride content detection in porcine preadipocytes. Our results demonstrated a significant decrease in lipid droplet accumulation and triglyceride content in the overexpression group compared to the negative control group from the 8th to the 12th day (Fig. 5A-B). We further quantified the concentration of fatty acid synthase using an ELISA kit and found a similar decreasing trend in fatty acid synthase levels (Fig. 5C, Additional file 2: Fig. S6). Building upon these findings, we noted that the mRNA levels of *PPARγ* and *FABP4* in the overexpression group were significantly lower than those in the negative control group on the 8th, 10th, and 12th day (Fig. 5D-F). Additionally, on the 8th day, we observed down-regulation of genes related to fatty acid synthase and the Wnt/β-catenin signaling pathway in the overexpression group. Similarly, we compared the differences between the RNAi group and the negative control group. The results of oil red O staining (Fig. 5G) and triglyceride content detection (Fig. 5H) also demonstrated that silencing the *DKK2* promoted adipogenesis. Furthermore, we observed a significant up-regulation of mRNA levels of *PPARγ* and *FABP4* in the RNAi group on the 8th and 10th day compared to the negative control group (Fig. 5I-J).

In 3T3-L1 cells, we observed similar effects when overexpressing *DKK2*, including the down-regulation of lipid droplet aggregation and triglyceride content (Additional file 2: Fig. S7A-B), as well as a decrease in fatty acid synthase concentration (Additional file 2: Fig. S7C). Additionally, we observed a decrease in the expression levels of relevant genes during the 4th to 8th day (Additional file 2: Fig. S7D-F) when *DKK2* was overexpressed. Conversely, silencing *DKK2* had opposite effects on the 4th day (Additional file 2: Fig. S7G-I). Collectively, our data supports the conclusion that *DKK2* inhibits fatty acid synthesis and adipogenesis through the Wnt/beta-catenin signaling pathway, acting as an inhibitor.

## Discussion

In this study, we conducted whole genome resequencing on 55 individuals from the Dabasha crossbred line and obtained whole genome sequencing data for an additional 12 pigs from a public database for analysis. PCA, phylogenetic tree and population structure revealed a consistent genetic pattern of pedigree. Additionally, we observed that the Shaziling pig population exhibited high genetic diversity, which may be attributed to the minimal artificial selection pressure it has experienced. Interestingly, our analysis revealed that the DBS population had a higher degree of genetic similarity with the Y population compared to other populations. This suggests that the DBS population may have received a greater genetic influence from the Y population.

By performing genome-wide selection scan, we identified 111 Shaziling unique selected genes, and we found several promising pathways related to the traits we focus on after annotation. Obviously, the identified phospholipid binding showed the strongest enrichment statistical signal (adjust *P* value = 0.002148), with 4 positive selection related genes which are also enriched in the term of lipid binding involved (*PIGU*, *MAP1LC3A*, *SNX24*, *SNX2*). *PIGU* was reported to be mostly related to diseases and cancers and also affects metabolism and endocrine of body [47, 48], which may be the reason of Shaziling pigs’ better disease resistance; *MAP1LC3A* may be related to the size and reproductive capacity of pigs [49], while it was reported that *SNX24* and *SNX2* may be associated with the content of trace elements in muscle [50]. KEGG analysis showed that 5 promising genes were enriched in fatty acid degradation (*ADH1C*, *LOC100512795*, *ADH4*, *ADH5*, *ACSL6*). *ADH1C* has been reported to impact on intramuscular fat, muscle marbling and the content of vitamins [51]; *ADH* gene family may be relevant to the fatty acid content in pork [52]; Previous study also reported that *ACSL6* could affect growth performance and meat quality traits of pigs via adjusting lipid synthesis [53]. The pathway of retinol metabolism was another promising enrichment pathway whose candidate genes were duplicated with fatty acid degradation. Retinol, also known as vitamin A1, is a fat-soluble vitamin that plays a vital role in various physiological functions for animals, including promoting growth and reproduction [54]. In pigs, studies have shown that the removal of vitamin A from their diet can lead to increased intramuscular fat and other metabolic alterations [55]. Furthermore, the withdrawal of dietary vitamin A has been found to enhance the accumulation of vitamin E [56], which has positive effects on meat quality characteristics [57]. Vitamin E is commonly used to improve meat stability and shelf life due to its ability to prevent lipid oxidation [58]. Thyroid peroxidase (*TPO*) was also a promising selected gene which encodes a membrane-bound glycoprotein enzyme and plays a central role in thyroid gland function. Previous studies have reported that the expression levels of *TPO* in pig may have a positive relationship with the weight gain and intramuscular fat content [59]. All in all, we identified some possible relevant pathways and candidate genes which may affect meat quality which could be further studied.

Building upon this, we conducted an IBD analysis to trace the transmission of 9 selected genes mentioned earlier from Shaziling pigs to Dabasha pigs. Among these genes, dickkopf WNT signaling pathway inhibitor 2 (*DKK2*) emerged as the most promising candidate. *DKK2* acts as an antagonist of the Wnt/beta-catenin signaling pathway. Currently, research on *DKK2* primarily focuses on tumors, with several papers reporting abnormal expression of *DKK2* in tumor tissues [60–62]. Furthermore, studies have indicated that the SNP (C29T) in exon 1 of the *DKK2* is associated with intramuscular fat content in Qinchuan cattle [63]. In experiments using c3h/10t1/2 mesenchymal stem cells of mice, researchers have verified that the *DKK2* can inhibit lipogenesis [64]. Additionally, research has shown that deficiency in *DKK2* expression leads to increased liver glycogen accumulation and decreased liver glucose output, suggesting that *DKK2* may influence the function of adipose tissues [65]. Moreover, numerous studies have highlighted the effect of WNT signaling on adipogenesis [66]. Given that the *DKK* family acts as inhibitors of the WNT signaling pathway, they may have a significant relationship with carcass traits and meat quality in pigs. This highlights a potential direction for further research in our study. In addition, we conducted an analysis of selective sweep signals for the *DKK2*. The results revealed that compared to Chinese local pigs, the *DKK2* in the S breed is subject to specific positive selection. Although both the S and European pig breeds showed positive selection signals, the Fst value results indicate that different selection pressures may have led to the selection of different haplotypes for the *DKK2* between the S and European pig breeds. These findings suggest that the *DKK2* in the S breed experiences different selective pressures and directions compared to both Chinese and foreign pig breeds, highlighting the unique genetic characteristics of the S breed.

In our study, we found that *DKK2* is associated with the content of palmitic acid and palmitoleic acid in pigs. This suggests that *DKK2* may influence meat quality through its effects on fatty acids. Palmitic acid, also known as hexadecanoic acid, is a high-grade saturated fatty acid that is commonly found in nature. It is present in varying amounts in different oils. Palmitic acid is one of the main saturated fatty acids (24% ~ 26%) in pig ketone bodies [67]. It is also a key flavor component in pork and has a significant impact on meat quality. On the other hand, palmitoleic acid, also known as soft-shelled turtle acid, is a monounsaturated fatty acid. It ranks fourth in terms of abundance (around 3%) in pig muscle. Studies have shown that palmitoleic acid contributes to the improvement of carcass traits, meat quality, and nutritional value in meat [68].

The SNP (g.114874954 A > C) site identified in this study showed that the A allele frequency of the SNP site was significantly negatively correlated with intramuscular fat content. Among them, only the Y was an exception. As it has a low intramuscular fat content but has the advantage frequency of C allele, whose genotype was similar to Chinese local pigs, which may be the result of crossbreeding with Meishan pigs for improvement [12]. Although it was detected as an intron SNP which does not cause changes in the encoded amino acids, previous studies have indicated that SNPs of intron have influence on the translation process by affecting alternative splicing or acting as enhancers to drive the expression of genes lacking promoters [69], especially the SNPs in the first intron may be more likely to affect the efficiency of mRNA splicing [70, 71]. Moreover, several studies have reported that SNPs in intron were found relevant to production traits in pigs (such as litter size, backfat thickness, etc.), which provided theoretical support and potential direction for further research [72–74]. This locus can predict the intramuscular fat content of pigs in an early, rapid, low-cost, and effective way, which could be used as a useful molecular marker for genetic breeding improvement of pigs.

Eventually, we preliminarily identified that *DKK2* gene plays a similar role in porcine preadipocytes and 3T3-L1 cells based on the technologies of overexpression and RNA interfere*.* In the middle and late stages of adipogenic differentiation, compared to the control group, the content of fatty acid synthase and the expression of adipogenic factors (*PPARγ, FABP4*) of negative group were significantly inhibited, which is consistent with the reported research results on mouse c3h/10t1/2 mesenchymal stem cells [64]. *PPARγ* (Peroxisome proliferators-activated receptors γ), located at the core of the C/EBP α adipogenesis pathway, is the main regulator of adipogenesis. It controls a variety of differentiation-dependent target genes, such as *aP2*, *CD36*, *LPL*, *PEPCK*, which play critical roles in the uptake and storage of triglycerides [75, 76]. *FABP4* (Fatty acid-binding protein 4) is one of the most abundant fat marker factors in adipocytes, which activates receptors by interacting with hormone-sensitive lipase (HSL) and *PPARγ* interaction, which plays an important role in maintaining adipocyte homeostasis, regulating adipolysis and adipogenesis [77, 78]. Furthermore, we observed changes in the mRNA levels of β-catenin (*CTNNB1*) and TCF/LEF (*TCF4*), suggesting that the *DKK2* may play a role through the classical Wnt signaling pathway. It has been demonstrated that the Wnt/β-catenin signaling pathway plays a role in adipocyte differentiation. The TCF/LEF complex could recruit transcriptional co-activators that are involved in the initiation of fat differentiation [66]. Based on the expression profile of *DKK2* and the aforementioned information, we can infer that the *DKK2* may play a role in the later stage of fat deposition. It likely acts to inhibit excessive fat accumulation and maintain intramuscular fat content within an appropriate range. In summary, our results indicated that the *DKK2* has a negative regulatory effect on adipogenic differentiation through the Wnt signaling pathway, which may be the way it affects meat quality.

## Conclusion

This study represents the first comprehensive analysis of the Shaziling pig population and other pigs in the Dabasha three-way crossbred line using whole genome resequencing. Through an integrated analysis of genome-wide selective sweep and identity-by-descent (IBD) analysis, we identified 9 novel genes that are not only inherited from Shaziling pigs to Dabasha pigs but also specifically selected in Shaziling pigs. Among these genes, *DKK2* was found to be associated with fatty acid content. Specifically, we discovered a mutation (A > C) at position 12,726 within intron 1 of the *DKK2*, which was found to be significantly associated with intramuscular fat content. Further investigation revealed that *DKK2* is specifically expressed in adipose tissue and has inhibitory effects on the adipogenic differentiation of porcine preadipocytes and 3T3-L1 cells, as well as fatty acid synthesis, ultimately impacting meat quality. Our findings provide valuable insights into the unique genetic characteristics of Shaziling pigs and highlight their potential as valuable genetic resources for improving pig meat quality.

### Supplementary Information

**Additional file 1: Table S1. **Primers and annealing temperatures used for PCRs in this research. **Table S2.** shRNA/siRNA target site of *DKK2 *gene. **Table S3.** Population size and resequencing depth of four pig breeds. **Table S4.** Quality control, mapping rate and coverage depth of 55 resequenced samples and 12 downloaded samples. **Table S5.** Results of SNP calling and annotation. **Table S6.** Statistics of common SNVs in each population. **Table S7.** Corresponding cross-validation error of presumed ancestral population. **Table S8.** Shaziling pigs' unique selected candidate genes. **Table S9.** GO (MF) analysis of S unique selected genes. **Table S10.** KEGG pathway analysis of S unique selected genes. **Table S11.** IBD analysis via S-BS-BS. **Table S12.** GO enrichment of candidate genes of IBD analysis via S-BS-DBS. **Table S13.** KEGG enrichment of candidate genes of IBD analysis via S-BS-DBS. **Table S14.** Resequencing data of downloaded Individuals for *DKK2* selective swwep analysis. **Table S15.** Selective sweep analysis of Shaziling pigs and other Chinese local pigs. **Table S16. **Selective sweep analysis of foerign pigs and Chinese local pigs. **Table S17.** Phenotypic traits data of Dabasha crossbred line. **Table S18.** SNP varitation of *DKK2* gene.

**Additional file 2: Fig. S1. **The interfere effect of shRNA (A) and siRNA (B) and its optimum transfection concentration 60 pmol. **Fig. S2. **Population Structure of All Individuals by Admixture (K= 2 to 12) and corresponding cross-validation error of presumed ancestral population. **Fig. S3. ***DKK2* gene sequence part comparison of 87 individuals of 16 populations. **Fig. S4. A** Tissue expression profile of Duroc and Shaziling pigs. **B** Relative expression of *DKK2* in abdominal fat and backfat of Duroc and Shaziling pigs. **Fig. S5. **Spatiotemporal expression profiles analysis of *DKK2* gene. **A ***DKK2* gene expression profile of porcine preadipocytes after 12 days’ differentiation.** B ***DKK2* gene expression profile of 3T3-L1 cells after 8 days’ differentiation. **Fig. S6. **Fatty acid synthase concentration standard curve of pig (A) and mouse (B). **Fig. S7. **The overexpression and RNA interfere of* DKK2* gene on 3T3-L1 cells.

## Data Availability

The authors declare that the data supporting the findings of this study are available within the article and its supplementary information files. All the raw sequences have been deposited in the NCBI database Sequence Read Archive with the BioProject number PRJNA988454.

## Abbreviations

WGS: Whole Genome Sequencing
SNP: Single Nucleotide Polymorphism
UTR: Untranslated Region
PCA: Principal Component Analysis
InDel: Insertion-Deletion
QTL: Quantitative Trait Locus
IBS: Identity by State
IBD: Identity by Descent
NJ: Neighbor Joining
LD: Linkage Disequilibrium
GO: Gene Ontology
KEGG: Kyoto Encyclopedia of Gene and Genomes
