# Supplementary material for: Integrated analysis strategy of genome-wide functional gene mining reveals DKK2 gene underlying meat quality in Shaziling synthesized pigs
Source: BMC Genomics. 2024 Jan 4;25:30. doi: 10.1186/s12864-023-09925-x (PMC10765619; doi:10.1186/s12864-023-09925-x)
Supplement: Supplementary file 2 — Additional file 2: Fig. S1. The interfere effect of shRNA (A) and siRNA (B) and its optimum transfection concentration 60 pmol. Fig. S2. Population Structure of All Individuals by Admixture (K= 2 to 12) and corresponding cross-validation error of presumed ancestral population. Fig. S3. DKK2 gene sequence part comparison of 87 individuals of 16 populations. Fig. S4. A Tissue expression profile of Duroc and Shaziling pigs. B Relative expression of DKK2 in abdominal fat and backfat of Duroc and Shaziling pigs. Fig. S5. Spatiotemporal expression profiles analysis of DKK2 gene. A DKK2 gene expression profile of porcine preadipocytes after 12 days’ differentiation. B DKK2 gene expression profile of 3T3-L1 cells after 8 days’ differentiation. Fig. S6. Fatty acid synthase concentration standard curve of pig (A) and mouse (B). Fig. S7. The overexpression and RNA interfere of DKK2 gene on 3T3-L1 cells. [file 12864_2023_9925_MOESM2_ESM.pdf]

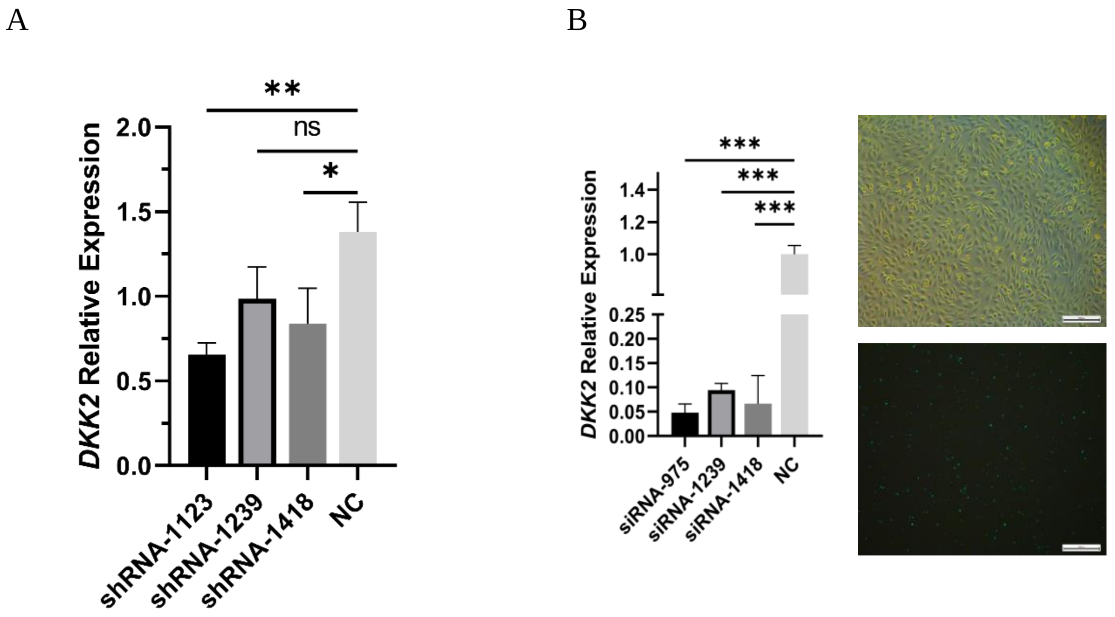

Fig. S1 The interfere effect of shRNA (A) and siRNA (B) and its optimum transfection concentration 60 pmol.

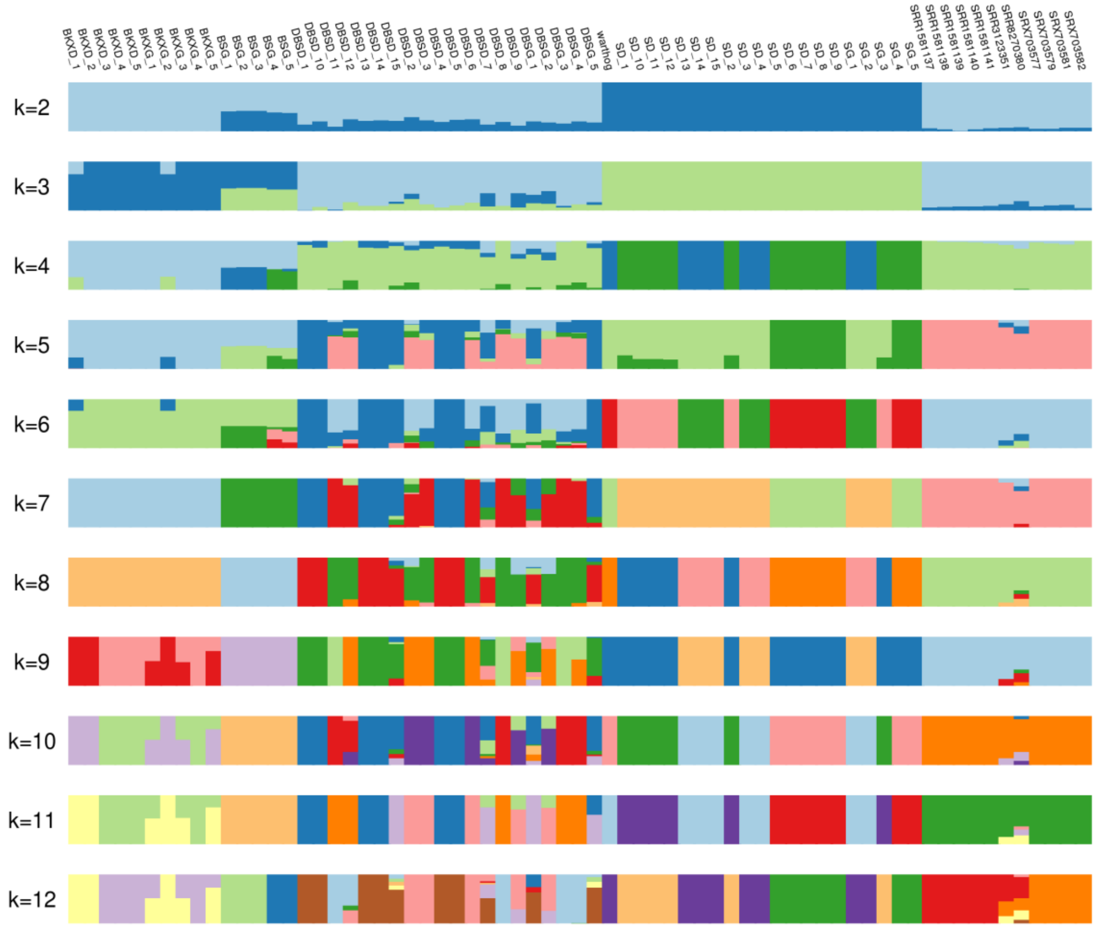

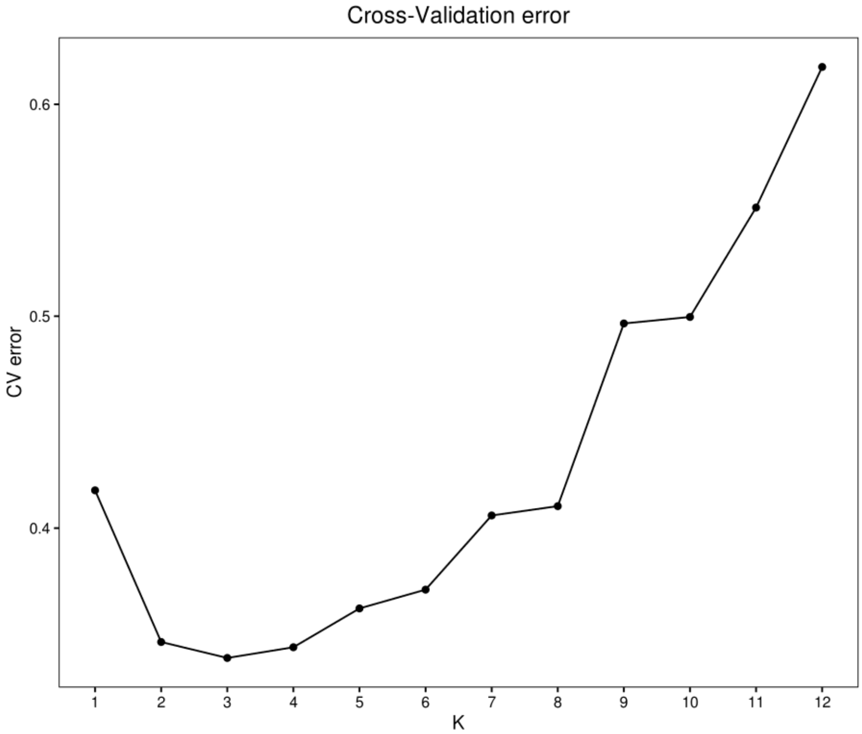

Fig. S2 Population Structure of All Individuals by Admixture (K= 2 to 12) and corresponding cross-validation error of presumed ancestral population.

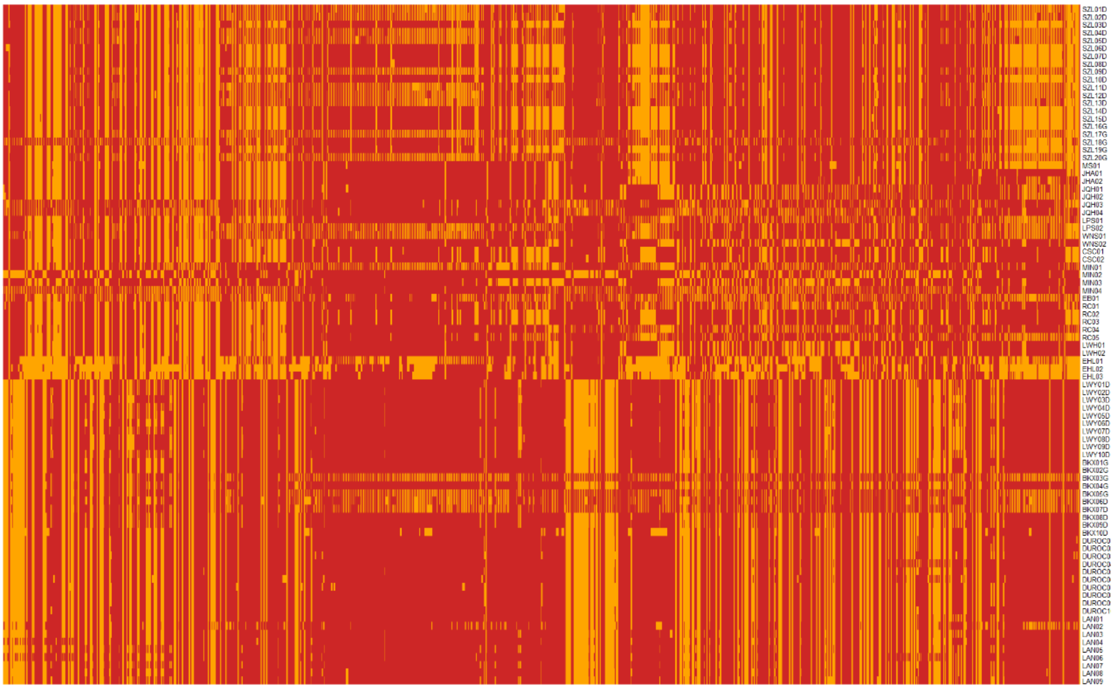

Fig. S3 *DKK2* gene sequence part comparison of 87 individuals of 16 populations. Find the specific information of individual name and breed in (Additional file 1: Table S4&14).

A B

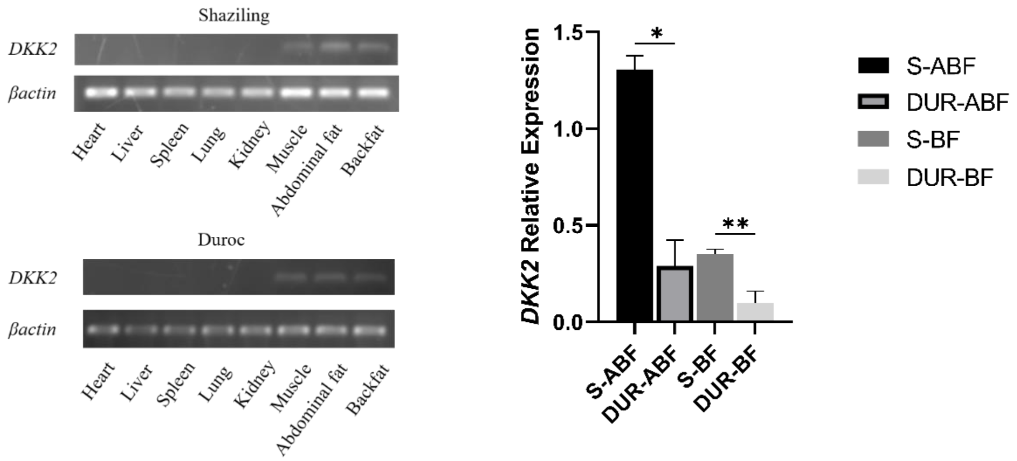

Fig. S4 **A** Tissue expression profile of Duroc and Shaziling pigs. **B** Relative expression of *DKK2* in abdominal fat and backfat of Duroc and Shaziling pigs.

A B

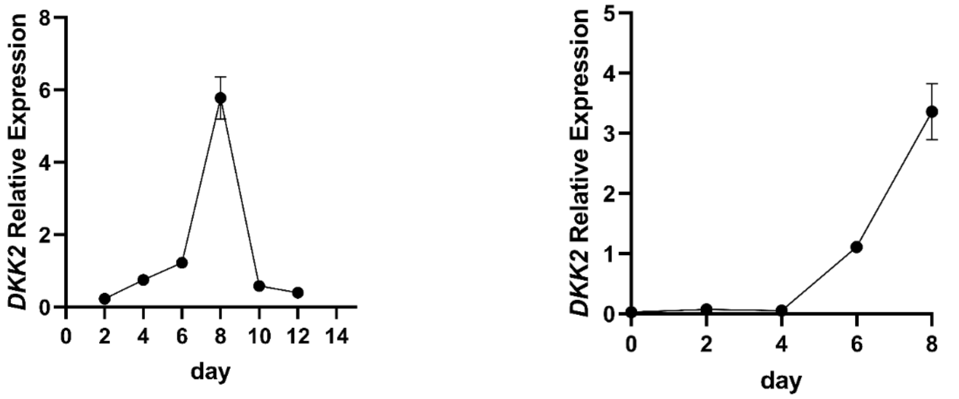

Fig. S5 Spatiotemporal expression profiles analysis of *DKK2* gene. **A** *DKK2* gene expression profile of porcine preadipocytes after 12 days' differentiation. **B** *DKK2* gene expression profile of 3T3-L1 cells after 8 days' differentiation.

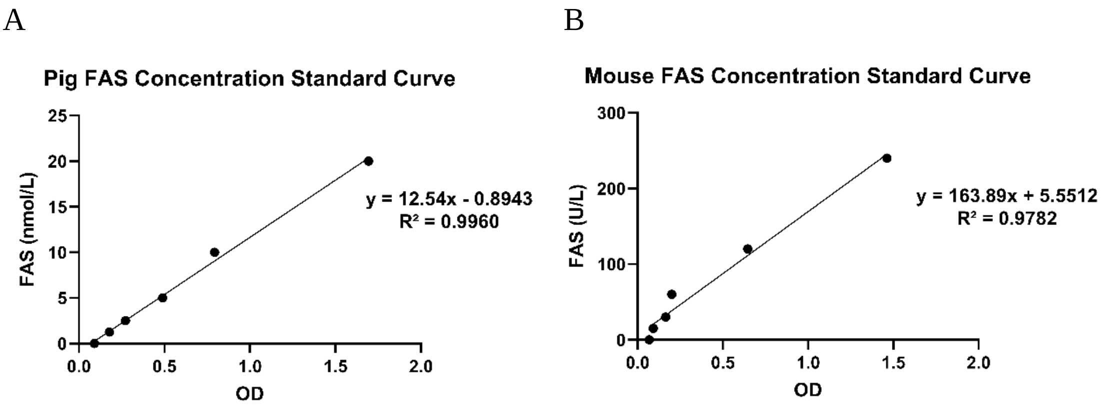

Fig. S6 Fatty acid synthase concentration standard curve of pig (A) and mouse (B).

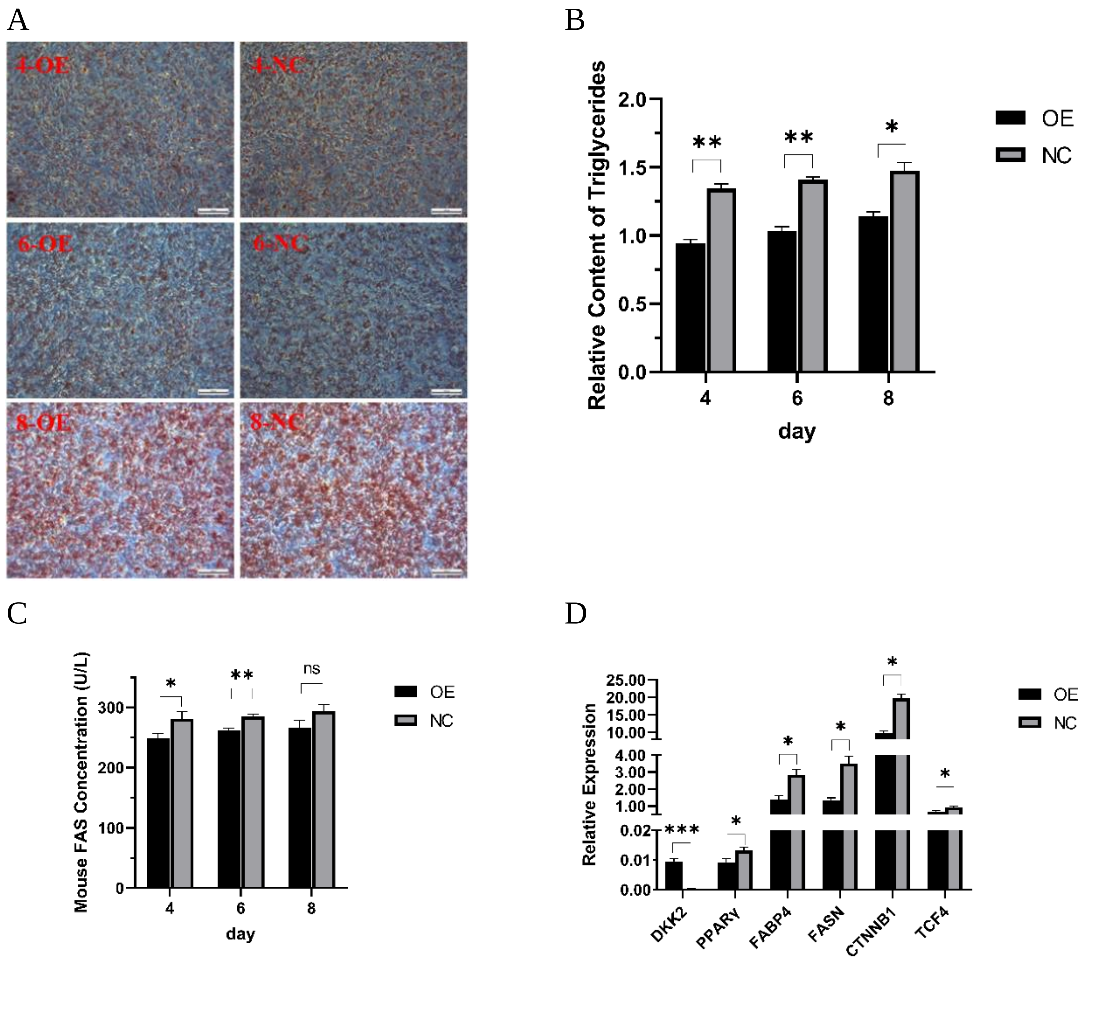

E

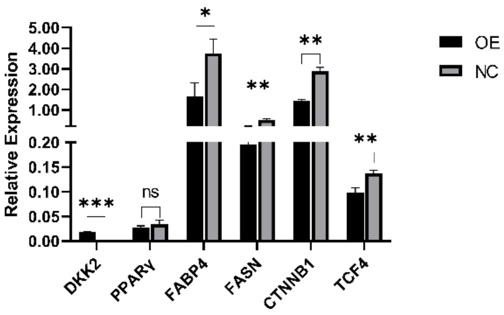

F

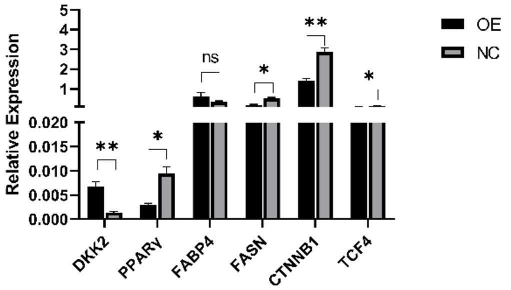

G

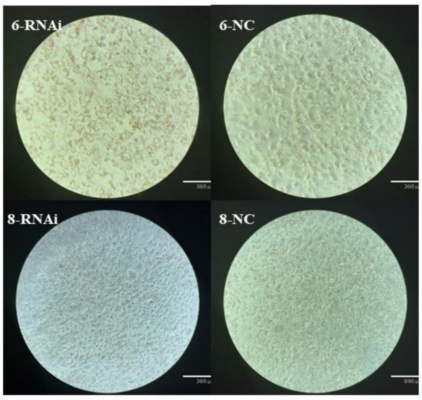

H

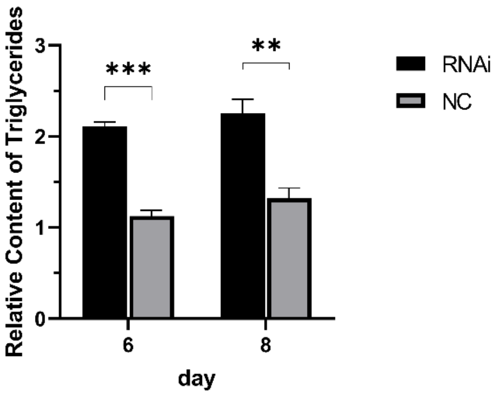

I

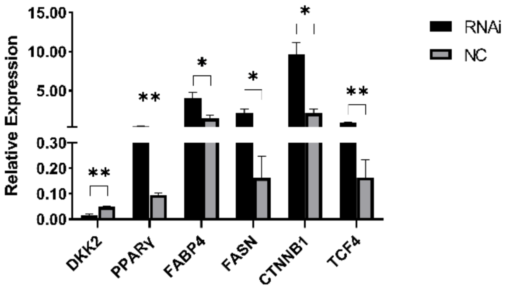

Fig. S7 The overexpression and RNA interfere of *DKK2* gene on 3T3-L1 cells**.** The mRNA levels of overexpression group and negative control group on 4th (**A**), 6th(**B**), 8th(**C**) day after differentiation. **D** Overexpression and negative control group's concentration of fatty acid synthase on 4th, 6th and 8th day after differentiation. **E** Lipid droplet accumulation of overexpression and negative control group on 4th, 6th and 8th day after differentiation. **F** Relative content of triglycerides of overexpression and negative control group on 4th, 6th and 8th day after differentiation. **G** The mRNA levels of RNAi group and negative control group on 4th day. **H** Lipid droplet accumulation of overexpression and negative control group on 6th and 8th day after differentiation. **I** Relative content of triglycerides of overexpression and negative control group on 6th and 8th day after differentiation.
